# Supplementary material for: Inverse design of 3D nanophotonic devices with structural integrity using auxiliary thermal solvers
Source: Nanophotonics. 2025 Apr 15;14(9):1415–26. doi: 10.1515/nanoph-2025-0067 (PMC12038610; doi:10.1515/nanoph-2025-0067)
Supplement: Supplementary file 1 — Supplementary Material Details [file j_nanoph-2025-0067_suppl_001.pdf]

Research Article

Oliver Kuster*, Yannick Augenstein, Roberto Narváez Hernández, Carsten Rockstuhl, and Thomas Jebb Sturges

Supplementary Material: Inverse Design of 3D Nanophotonic Devices with Structural Integrity using Auxiliary Thermal Solvers

1 Optimized structures without a heat solver

Here we show the respective optimization results without using an auxiliary heat solver. In both cases our figure of merit is $\mathscr{L} = -\mathscr{L}_{\mathrm{EM}}$, only optimizing for the optical performance of the designs. Other than that, the setups are exactly the same as described in the main text

1.1 Focusing Device

Figure 1 shows the optimized design. It can be clearly seen, that the center of the design is free floating, without any additional support structures. What cannot be seen is, that the design also contains cavities. These cavities can be seen in the animations provided in the additional supplementary material.

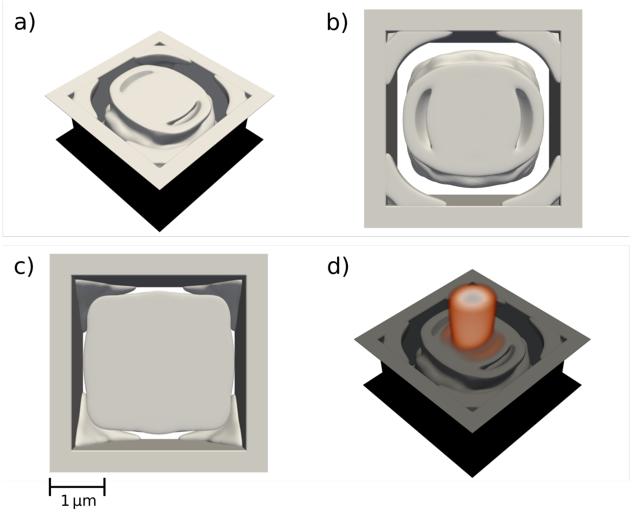

Fig. 1: a)-c) optimized focusing device without the heat constraints from different angles. d) shows the optimized design with the enhanced electric field distribution.

1.2 Waveguide Coupler

Figure 2 shows the optimized design. While the majority of the design is structurally integral, some free floating parts appear. The design does not contain any cavities, which can also be seen in the animations provided in the supplementary material. It should still be noted, that this design uses more material than a design which uses the auxiliary heat solver, while achieving minimally improved performance.

2 Animations

We provide several animations, which show the optimized designs with and without the auxiliary heat solver. These can be found under https://github.com/OlloKuster/Structural_Integrity_3D/tree/main/Animations. 16 different animations are shown. First 2 for two directions are given and denoted by "_x/y". Then we have simulations for the "material" and "void" each and for "opt" and not, meaning optimized using an auxiliary heat solver and without an auxiliary heat solver. Lastly, we provide "heat" plots, which show $u(x,y,z)$ in addition to the structure. Note, that the material/void plots are done using

***Corresponding author: Oliver Kuster,** Institute of Theoretical Solid State Physics, Karlsruhe Institute of Technology (KIT), Kaiserstrasse 12, 76131 Karlsruhe, Germany, oliver.kuster@kit.edu; https://orcid.org/0009-0003-2717-7690
Yannick Augenstein, Flexcompute Inc, Belmont, MA, USA
Roberto Narváez Hernández, Institute of Theoretical Solid State Physics, Karlsruhe Institute of Technology (KIT), Kaiserstrasse 12, 76131 Karlsruhe, Germany
Carsten Rockstuhl, Institute of Theoretical Solid State Physics, Karlsruhe Institute of Technology (KIT), Kaiserstrasse 12, 76131 Karlsruhe, Germany and Institute of Nanotechnology, Karlsruhe Institute of Technology (KIT), Kaiserstrasse 12, 76131 Karlsruhe, Germany; https://orcid.org/0000-0002-5868-0526
Thomas Jebb Sturges, Institute of Theoretical Solid State Physics, Karlsruhe Institute of Technology (KIT), Kaiserstrasse 12, 76131 Karlsruhe, Germany;

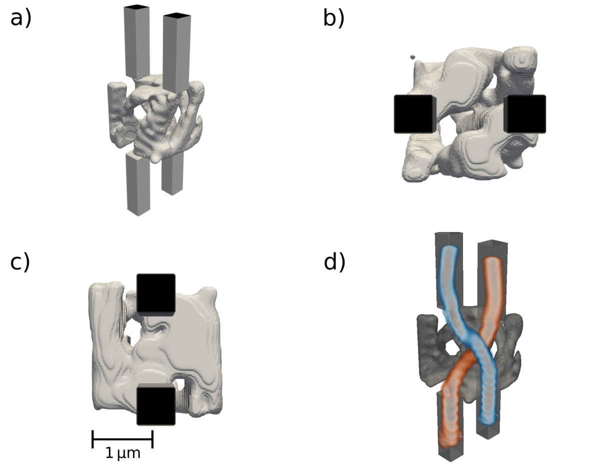

Fig. 2: a)-c) optimized waveguide coupling device without the heat constraints from different angles. d) shows the optimized design with the enhanced electric field distribution.

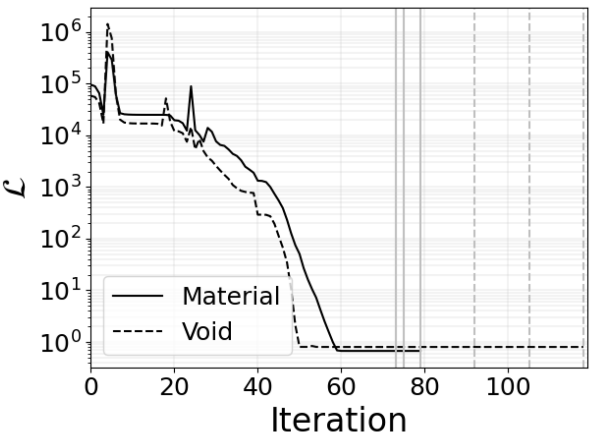

Fig. 3: Two optimizations of a focusing device. The optimization was conducted until convergence for a low threshold value, leading to a trivial solution (roughly after 60 iterations). Then the optimization is continued from the previously found local minimum by increasing the threshold values. Each vertical line indicates an increase of the threshold value by two orders of magnitudes. We see that the optimizer stays in the initially found solution and is not able to escape its local minima and stays at the trivial solutions.

contour plots. The designs themselves are not hollow, but for better visualization, we use the contour plots.

3 Probing the local minima

To get a deeper understanding of how the figure of merit works, we look into the behavior of the optimization. For simplicity, we only look at the focusing device, as the transition between the trivial structures and non-trivial structures is seen more easily in this example.

First, we use a very small threshold value $\mathscr{L}_{\text{heat}}^{\text{thresh}}$. The optimizer finds a trivial structure that consists of only material or only void (see red and black square in Fig. 2 of the main manuscript respectively). Once we have converged (which happens roughly after 60 iterations), we increase the threshold value $\mathscr{L}_{\text{heat}}^{\text{thresh}}$ and optimize the device from that initially obtained solution. We repeat this process a few times. The results of these optimizations can be seen in Fig. 3. The vertical lines mark the points where we increased the threshold value by two orders of magnitudes. After each increase, we kept the optimization going until convergence (as stated in the main text). As we do know that there are non-trivial solutions for higher threshold values, i.e., they were obtained by optimizing the designs for each given threshold value from scratch, we know that their respective local minima do exist for the figure of merit. Nonetheless, the optimization is unable to reach local minima which represent a functional optical device and is stuck in the local minimum it converged towards initially. While the optimization shown in Figure 3 is able to reach values of $\mathscr{L} \approx 0.8$, the optimization mostly optimized for the thermal performance where we start with $\mathscr{L}_{\text{heat}}^{n} \approx 6000$, whereas $\mathscr{L}_{\text{EM}}^{n} \approx 0.6$ for for the initial simulation. Ideally, we want to reach $\mathscr{L} \leq 0.2$ for a well functioning, structurally connected device. By optimizing for the thermal performance first, the electromagnetic sub-objective stays relatively constant throughout the entire optimization, or can get even worse, leading to the trivial solutions shown in the main manuscript.

This suggests that we cannot escape the local minimum once we have converged towards it, even if there is a more suitable one available somewhere else. However, the problem is circumvented by optimizing the devices from scratch for every considered threshold value, as done in the main manuscript.

Secondly, we look at which local minima are found with randomly initialized density distributions. To do so, we repeat the procedure detailed in the main text, but instead of using a uniform density distribution, we use seeded, randomly initialized density distributions. Figure 4 shows the best found $\mathscr{L}_{\text{EM}}$ for different threshold values $\mathscr{L}_{\text{heat}}^{\text{thresh}}$. Overall, almost no difference between the different randomly initialized and the uniformly initialized distribution can be found.

4 Resolution of the simulation

To verify if our chosen spatial resolution in the simulations is sufficient, the simulations are run at a lower resolution with the optimized structures, and we analyze the convergence behav-

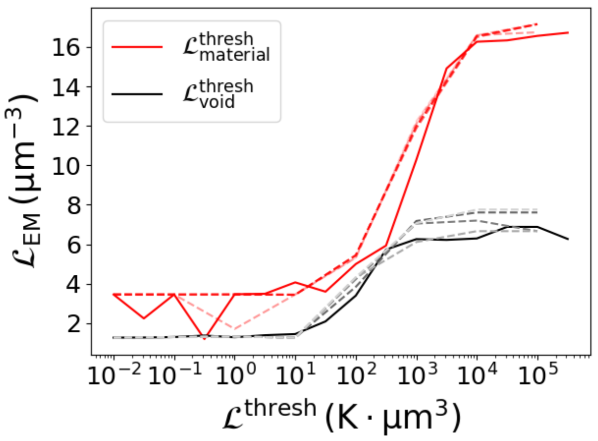

Fig. 4: Dependence of $\mathscr{L}_{\text{EM}}$ with respect to the threshold value of the heat problem. The red curves show the dependence of $\mathscr{L}_{\text{EM}}$ on $\mathscr{L}^{\text{thresh}}_{\text{material}}$ with a fixed void threshold value. The black-gray curves show the dependence of $\mathscr{L}_{\text{EM}}$ on $\mathscr{L}^{\text{thresh}}_{\text{void}}$ with a fixed material threshold value. Various randomly initialized density distributions are shown as dashed lines. The solid lines present the dependence as shown in the main manuscript with a density distribution which was initialized with a uniform distribution.

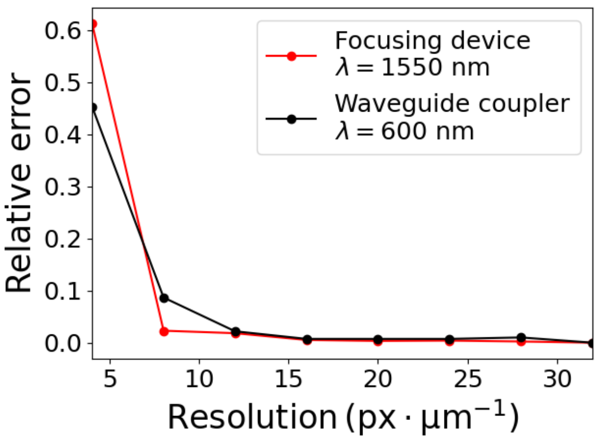

Fig. 5: Evaluation of the impact of the spatial resolution in the FDFD simulation on the results. The figure shows the relative error of the electromagnetic objective function of the focusing device and the waveguide coupler as shown in main manuscript depending on the spatial resolution considered in the FDFD simulations. Indeed, we observe convergence already for a resolution of roughly ten pixels per micrometer. This is in agreement with the common expectation of dielectric materials in FDFD for the considered wavelengths.

ior of the electromagnetic objective function depending on the resolution. The results of these additional simulations can be seen in Fig. 5, where we changed the spatial resolution in terms of pixel per micrometer in our finite-difference finite-domain (FDFD) simulations. Here, we study the impact of the spatial resolution considering the most optimal focusing device, i.e., the focusing device shown in Fig. 3 of the main manuscript and the optimal waveguide coupler shown in Fig. 5 of the main manuscript. We evaluated the electromagnetic figure of merit as described in the main text but changed the spatial resolution at which the optical simulations are performed as well as the resolution of the considered device. We then compare the relative error of the resulting electromagnetic figure of merit

$$E = \frac{|\mathscr{L}^{\text{opt}}_{\text{EM}} - \mathscr{L}^{\text{current}}_{\text{EM}}|}{\mathscr{L}^{\text{opt}}_{\text{EM}}}, \tag{1}$$

where $\mathscr{L}^{\text{opt}}_{\text{EM}}$ refers to the figure of merit the structure at $40\,\text{px}\cdot\mu\text{m}^{-1}$ evaluates at and $\mathscr{L}^{\text{current}}_{\text{EM}}$ refers to the figure of merit of a structure with the respective lower resolution. We note, that we only evaluated the waveguide coupler at 600 nm wavelength, as a sufficient resolution at 600 nm would also be sufficient for 700 nm. Additionally, The figure of merit considered in the main manuscript is not independent of the resolution. So to properly analyze the convergence behavior depending on the resolution, we renormalize the electromagnetic figure of merit for the waveguide coupler by the crossectional area of the waveguide $\mathscr{L}^{\text{renorm}}_{\text{EM}} = \frac{\mathscr{L}_{\text{EM}}}{w^2}$, where w is the width of the waveguide.

Clearly, the resulting error converges already right around $10\,\text{px}\cdot\mu\text{m}^{-1}$ in both cases. This is not surprising, considering the fact that the polymer materials that are considered in the structure have a rather low permittivity and are on their own optically not that complicated to evaluate.

5 Non-trivial solutions for the waveguide coupler

Fig. 6 shows solutions to the waveguide coupling problem which do not consist of only void and are thus non-trivial. These solutions appear before and right around the sharp increase in performance seen in Fig. 4 of the main manuscript for the sweep of $\mathscr{L}^{\text{thresh}}_{\text{material}}$. Still, these solutions (with the exception of d)) are not able to meaningfully guide the light to the outgoing waveguides. Since the heat sinks sit on all of the four waveguides, structures which do not connect the ingoing to the outgoing waveguide can be found which is why they do not qualify as fully connected in a strict sense.

6 Softplus function

Figure 7 shows the *softplus* function in the range $[-1, 1]$. Our renormalization is done in a way, that -1 denotes the low-

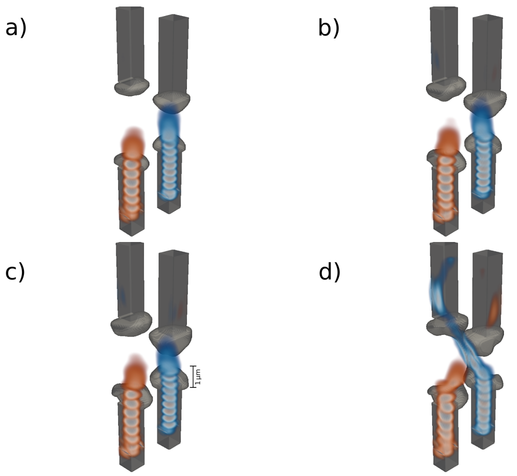

Fig. 6: a) - c) Different solutions found for the waveguide coupler which are not completely trivial (e.g. only void) but have little to no influence on the electromagnetic performance. d) shows a device where one of the ingoing waveguides is connected to its outgoing waveguide, but not both. The threshold values used are a) $\mathscr{L}^{\text{thresh}}_{\text{material}} = 100\,\text{K} \cdot \mu\text{m}^3$, b) $\mathscr{L}^{\text{thresh}}_{\text{material}} = 251\,\text{K} \cdot \mu\text{m}^3$, c) $\mathscr{L}^{\text{thresh}}_{\text{material}} = 398\,\text{K} \cdot \mu\text{m}^3$ and d) $\mathscr{L}^{\text{thresh}}_{\text{material}} = 631\,\text{K} \cdot \mu\text{m}^3$ and $\mathscr{L}^{n}_{\text{void}} = 1\,\text{K} \cdot \mu\text{m}^3$ for all of these examples.

est possible value, while the positive range is unbound. We start to interpolate linearly after $x = 0.4$ for simplicity. Values below $x = 0$ start to rapidly decay, leading to an optimization procedure which automatically puts emphasis on values which haven't crossed their chosen threshold value.

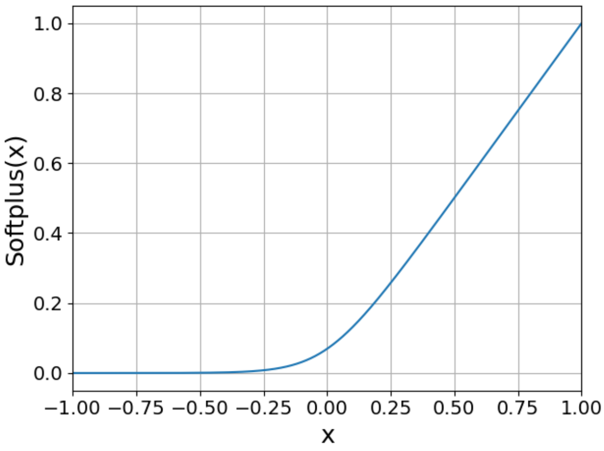

Fig. 7: Softplus function
